# Factors Associated with Suspected Developmental Delay in Thai Children Born with Low Birth Weight or Asphyxia

**DOI:** 10.1007/s10995-023-03814-1

**Published:** 2023-11-08

**Authors:** Duangkamol Tangviriyapaiboon, Vallop Thaineua, Samai Sirithongthaworn, Siripon Kanshana, Siriwat Damrongtamwattana, Sukon Prasitwattanaseree, Pimwarat Srikummoon, Natthapat Thongsak, Salinee Thumronglaohapun, Patrinee Traisathit

**Affiliations:** 1 Rajanagarindra Institute of Child Development, Chiang Mai, Thailand; 2 Department of Mental Health, Ministry of Public Health, Nonthaburi, Thailand; 3 Department of Health, Ministry of Public Health, Nonthaburi, Thailand; 4 Department of Statistics, Faculty of Science, Chiang Mai University, Chiang Mai, Thailand

**Keywords:** Pediatrics, Growth, Infant development, DAIM, Nutrition status, High-altitude area

## Abstract

**Objectives:**

The aim of the study was to identify factors associated with a risk of suspected developmental delay (SDD) in high-risk children in Thailand.

**Methods:**

We used data on children enrolled for developmental delay (DD) screening across Thailand collected by the Rajanagarindra Institute of Child Development, Department of Mental Health, Ministry of Public Health, Thailand. Children who were under 5 years of age with a birth weight of fewer than 2500 g and/or birth asphyxia in Thailand with high risk of DD were assessed using the Developmental Assessment for Intervention Manual (DAIM) between August 2013 and November 2019 (*N* = 14,314).

**Results:**

The high-risk children who had a gestational age at birth of < 37 weeks (adjusted odds ratio = 1.54; 95% confidence interval = 1.39–1.70) and/or had a birth weight < 2500 g (1.22; 1.02–1.45), or had mothers who were not government officers (1.46; 1.11–1.93), had a low education level (1.36; 1.19–1.55), had a poor nutritional status (1.34; 1.09–1.65), and/or who were living in a high-altitude area (1.59; 1.32–1.91) were at a higher risk of SDD.

**Conclusions for Practice:**

Children with a low birth weight and/or asphyxia during birth had a high risk of DD. SDD monitoring of children by community health workers and/or by developing outreach strategies, especially in underserved regions, should be considered. In addition, developing policies and guidelines, and intervention for high-risk children ought to be conducted to reduce the subsequent problems caused by the late detection of DD.

## Significance

*What is already known on this subject?* Developmental disability is a common problem in children under 5 years of age, especially in low-middle income countries. Previous studies reported that socio-economic and environmental factors (e.g., low household income, undernourishment, or living in an urban residence) and maternal factors (e.g., age during pregnancy, educational level, number of pregnancies, breastfeeding, or medical disorders during pregnancy, smoking, or drinking alcohol behavior) were associated with a higher risk of DD in children.

*What this study adds?* This study conducted a screening for suspected development (SDD) to reduce the potential negative effects of developmental delay. We also revealed the possible early identification of SDD by investigating the factors associated with SDD. Our findings suggested that a child born after a short gestation with low birth weight, having a mother who is not a government officer, has a low educational level, has poor nutritional status, and/or lives at a high altitude, are associated with a higher risk of SDD.

## Introduction

The initial formative years of life are the most important period for a child as during this time the brain develops quickly through neurogenesis, axonal and dendritic development, synaptogenesis, cell passing, synaptic pruning, myelination, and gliogenesis (Grantham-McGregor et al., 2007). Developmental disability is a common problem in children under 5 years of age. Globally, 52.9 million children younger than 5 years old have developmental disabilities. Of these children, around 95% live in low-middle income countries (Global Research on Developmental Disabilities Collaborators, 2018). In 2017, approximately 250 million children (43%) under 5 years of age living in low- and middle-income countries were estimated to be at risk of not achieving their full developmental potential (Black et al., 2017). Moreover, 15.1 million children (25.9%) aged 3–4 years old living in the East Asia/Pacific region had low developmental scores measured by using the Early Childhood Development Index (ECDI) (McCoy et al., 2016). In Thailand, developmental delay (DD) is still a long-standing problem, with the Ministry of Public Health reporting that 28.30%, 29.71%, and 26.60% of young children had uncommon development in 1999, 2010, and 2020, respectively (Kue-iad et al., 2018; The National Institute of Child Development, 2021). In addition, it has been reported that the prevalence of DD among Thai children aged under 5 years ranges from 27.2 to 30.3% (Chunsuwan et al., 2016; Losatiankit et al., 2017; Ratanatharathorn et al., 2022).

A previous study suggested that nearly a half of children (30–50%) who have developmental disorders are not identified until school age and so may not be treated early enough (Glascoe, 2000). Therefore, the early detection and appropriate referral of children with DD or disorder are important for pediatricians and behavioral psychologists to begin treating them.

Several screening tests have been created to measure child development. The Denver Developmental Screening Test II (Denver II) is an observational screening tool that has been commonly utilized in several countries (Eratay et al., 2015; Mirrett et al., 2004; Tanochni et al., 2015). However, in addition to the requirement of certification prior to administering it, it was developed for Western countries, so it may not be applicable to the Tahi context due to cultural differences. Thus, the Developmental Surveillance and Promotion Manual (DSPM) and the Developmental Assessment for Intervention Manual (DAIM) have been established for the assessment and monitoring of developmental milestones in Thai children from birth to 5 years old (Ratanatharathorn et al., 2022). Children with a high risk of DD (i.e., a birth weight of less than 2500 g and/or birth asphyxia (Apgar score ≤ 7 at 1 min)) are assessed by using the DAIM (Jundaboot et al., 2018; Kanpirom & Masena, 2017; Morrison et al., 2018; Saengjun, 2017; Vuttipittayamongkol, 2018) while children who are not at risk are assessed by using the DSPM (Ratanatharathorn et al., 2022).

It has been reported that socio-economic and environmental factors are associated with DD in children. The outcomes of several studies in low-middle income countries indicate that there is a higher risk of DD among children who come from a family with poor socio-economic status and/or with low household income, are undernourished, or live in an urban residence (Halpern et al., 2000; Losatiankit et al., 2017; Sitaresmi et al., 2016; Torabi et al., 2012; Zhang et al., 2018). The parent’s occupations are potentially associated with the risk of DD since it is directly linked to the household income but also indirectly linked to the mental health of the mother during pregnancy. Losatiankit et al. (2017) reported that children whose mothers were farmers had a higher risk of DD than those whose mothers were government officers (Losatiankit et al., 2017). Maternal factors are also associated with the risk of DD, such as age during pregnancy, educational level, number of pregnancies, breastfeeding, or medical disorders during pregnancy (Halpern et al., 2000; Miller et al., 2020; Sitaresmi et al., 2016; Torabi et al., 2012). Several researchers have reported that smoking or drinking alcohol during pregnancy can affect the development of the child (Hagan et al., 2016; Julvez et al., 2007; Slykerman et al., 2007; Yoshida et al., 2018). In addition, child-related factors such as low birth weight, a short gestation period, congenital heart disease, cyanosis, and/or a history of neonatal jaundice, bestow the child with a higher risk of DD (Halpern et al., 2000; Mussatto et al., 2014; Sitaresmi et al., 2016; Torabi et al., 2012). Living area is an environmental factor potentially associated with child development delay since it is representative of socio-economic status, quality of life, and healthcare access. The findings from previous studies in Thailand reveal that the people living in high-altitude areas suffer from a lack of education, healthcare, and nutrition (National Research Council of Thailand, 2016; Suwannasuan et al., 2013). In addition, exposure to air pollution in the high-altitude northern region of Thailand might increase the risk of brain harm and neurodevelopmental problems in children (Sunyer & Dadvand, 2019).

Due to the importance of the effect of DD on childhood morbidity, early detection of DD is necessary to reduce conceivable negative outcomes. Screening for suspected DD (SDD) after the first assessment is one of the early detection and prevention methods for DD. The child with SDD and their parents will be suggested to enroll in the promotion program for one month and followed-up for the confirmation diagnosis of DD at the second assessment (Ministry of Public Health, 2020). Surveillance of SDD and examining for its associated factors might be useful in monitoring and following-up for children who were at risk of DD. The aim of this study is to identify factors associated with SDD in high-risk children who were under 5 years of age with a birth weight of fewer than 2500 g and/or birth asphyxia in Thailand.

## Methods

### Study Design and Setting

This was a cross-sectional study conducted on high-risk children who were under 5 years of age and were at a high risk of DD (a birth weight of less than 2500 g and/or birth asphyxia) (Ratanatharathorn et al., 2022) across Thailand (332 hospitals in 36 provinces). The data were collected by the Rajanagarindra Institute of Child Development, Department of Mental Health, Ministry of Public Health, Thailand between 1 August 2013 and 11 November 2019.

### Assessment Tool

Children with a high risk of DD were assessed using the DAIM consisting of 137 items of which 130 cover for five developmental domains (gross motor skills, fine motor skills, receptive language skills, expressive language skills, and personal and social skills) and seven items for neurodevelopment (Ministry of Public Health, 2020). It was used to screen children soon after birth and at 1, 2, 3–4, 5–6, 7–8, 9, 10–12, 13–15, 16–17, 18, 19–24, 25–29, 30, 31–36, 37–41, 42, 43–48, 49–54, 55–59, and 60 months by trained health workers in Health Promoting Hospitals (HPHs). The DAIM had a sensitivity of 85.7% and a specificity of 86.3% compared to the Denver II (Phunsawat, 2016). Similar to the DSPM, the DAIM manual was designed to guide health workers and caregivers through the screening process by covering which activities the child should be able to do at their age within each developmental domain. The manual also has a pictorial guide and written instructions on how to practice the activities with the child (Morrison et al., 2018).

The health workers would review the characteristics of the child and their parents and familiarize them with the procedure prior to the start of screening. At the beginning of the screening process, the health workers would take the child to a private room free from outside distractions. The parents were allowed to be present in the room but had to avoid helping their child during the evaluation process. The health workers then commenced the screening process using the developmental section for children under 1 year old. For example, a child aged two months old was first evaluated using the one-month-old section. If the child passed, the health workers continued the testing using a higher age-range section and stopped when the child failed. If the child failed in the first stage, the health workers continued testing with a lower age-range section until the child passed the test. In the next developmental follow-up, testing started with the age-range section at which the child failed on the previous occasion. In the case of two tests for a particular age range, the child was noted as having failed if he/she failed either one of them (Ministry of Public Health, 2020). If the child failed to perform one of the activities, the caregiver was counseled, given the manual, and asked to return to the HPH on another visit to reassess the child. If the child failed the screening a second time, they were referred to an appropriate community or general hospital with specialist care (Morrison et al., 2018).

### Outcome

A child who underwent developmental screening by using the DAIM and failed to complete five domains in the first developmental assessment was diagnosed with SDD. Afterward, it would be suggested to the parents that the child be enrolled in a developmental promotion program for 30 days and followed up for a second assessment. Those who failed the second assessment were confirmed as having DD (Ministry of Public Health, 2020).

### Study variables

Information on maternal and child-related factors was collected in this study. The mothers were asked about their occupation [government officer, private business, company employment, or other (e.g., farmer, housekeeper, etc.)], educational level (primary school, junior high school, high school, college/university, or other), marital status (married, divorced, or other), the number of pregnancies, smoking status, alcohol consumption, illicit drug use, and living area. They were also asked about their pregnancies and children, including adverse pregnancy outcomes, gestational age at birth, child’s birth weight, congenital disorders in their children, and the Apgar score at 1 min after birth. Their answers were checked against their medical records where possible.

### Patient and Public Involvement

The participants only provided demographic information and took part in the evaluation of DD. They were not involved in other aspects of the research such as the study design, discussion of the outcomes, etc.

### Statistical Analyses

Baseline characteristics were presented as the median and interquartile range (IQR) for continuous variables and frequencies and percentages for categorical variables. Characteristics of the mother and child were included in the analyses: the mother’s marital status, occupation, education level, number of pregnancies, smoking status, nutritional status, alcohol consumption status, illicit drug use status, living at a high altitude, and/or had experienced an adverse pregnancy outcome, along with the child’s birth weight, gestational age at birth, congenital disease status, and health at birth. Outlier data were omitted using the following criteria: gestational age at birth < 20 weeks or > 43 weeks, birth weight < 500 g or > 5000 g, and no or less than eight pregnancies. Associated factors of SDD were assessed using binary logistic regression except for variables with more than 10% of missing data; the mother’s education level was nevertheless included in the analysis due to its potential effect on the development of the child. To control confounding and avoid bias when selecting variables, factors associated with the risk of SDD with *P* < 0.25 in the univariable analysis were included in the multivariable analysis with a backward elimination procedure (Mickey & Greenland, 1989). All analyses were performed using Stata version 12 (StataCorp, 2011).

## Results

### Baseline Characteristics

Of the 14,314 children included in the study, the number of children with SDD based on the DAIM assessment was 2733 (19.1%). The median (IQR) gestational age at birth and birth weight were 37 (34.93–38.00) weeks and 2290 (2020–2430) grams, respectively. Most of the children (87%) were at least 2500 g of birth weight with no history of asphyxia during birth. Most of the mothers had an educational level higher than primary school (86%) and were married (96%). Some of the mothers were exposed to potential risk factors for DD, including smoking (1%), poor nutritional status (4%), heavy alcohol consumption (0.2%), illicit drug use (0.3%), and living at a high altitude (5%) (Table 1).

**Table 1:** Baseline characteristics of the mothers and children in the study (N = 14,314)

Characteristics	n (%) or median (IQR)			*P*-value^a^
	All (N = 14,314)	Normal (n = 11,581)	SDD (n = 2733)	
Mother's occupation (m = 1035)				
Government officer	538 (4%)	467 (4%)	71 (3%)	< 0.001**
Private business	1112 (8%)	947 (9%)	165 (7%)	
Company employee	389 (3%)	335 (3%)	54 (2%)	
Other	11,240 (85%)	9095 (84%)	2175 (88%)	
Mother's educational level (m = 1924)				
Primary	1738 (14%)	1312 (13%)	426 (19%)	< 0.001**
Junior High School	3275 (26%)	2651 (26%)	624 (28%)	
High school	3609 (29%)	2975 (29%)	634 (28%)	
College/University	3337 (27%)	2840 (28%)	497 (22%)	
Other	431 (3%)	357 (4%)	74 (3%)	
Marital status (m = 1508)				
Married	12,280 (96%)	10,012 (96%)	2268 (96%)	0.485
Divorced	403 (3%)	323 (3%)	80 (3%)	
Other	123 (1%)	96 (1%)	27 (1%)	
Number of pregnancies (m = 856)				
1	6763 (50%)	5507 (50%)	1256 (50%)	0.521
2–3	5920 (44%)	4836 (44%)	1084 (44%)	
More than 3	775 (6%)	620 (6%)	155 (6%)	
Smoking status				
No	14,191 (99%)	11,473 (99%)	2718 (99%)	0.051
Yes	123 (1%)	108 (1%)	15 (1%)	
Poor nutritional status				
No	13,720 (96%)	11,138 (96%)	2582 (94%)	< 0.001**
Yes	594 (4%)	443 (4%)	151 (6%)	
Heavy alcohol consumption				
No	14,283 (99.8%)	11,555 (99.8%)	2728 (99.8%)	0.674
Yes	31 (0.2%)	26 (0.2%)	5 (0.2%)	
Illicit drug use				
No	14,270 (99.7%)	11,544 (99.7%)	2726 (99.7%)	0.590
Yes	44 (0.3%)	37 (0.3%)	7 (0.3%)	
High altitude				
No	13,639 (95%)	11,097 (96%)	2542 (93%)	< 0.001**
Yes	675 (5%)	484 (4%)	191 (7%)	
Had an adverse pregnancy outcome				
No	8912 (62%)	7191 (62%)	1721 (63%)	0.394
Yes	5402 (38%)	4390 (38%)	1012 (37%)	
Gestational age at birth (months; m = 1454)	37 (34.93–38)	37 (35–38)	36 (33–38)	< 0.001**
Birth weight (g; m = 1025)	2290 (2020–2430)	2310 (2070–2440)	2180 (1760–2390)	< 0.001**
Congenital disorder (m = 2031)				
No	11,980 (98%)	9792 (98%)	2188 (96%)	< 0.001**
Yes	303 (2%)	211 (2%)	92 (4%)	
Health at birth (m = 2031)				
Normal	10,662 (87%)	8780 (87%)	1882 (84%)	< 0.001**
Abnormal	1625 (13%)	1260 (13%)	365 (16%)	

*IQR* interquartile range, *N* number of all children, *n* number of children in each group; *%* percentages, *m* number of missing data items, *SDD* suspected developmental delay

^a^*P* values derived by using Chi-squared tests for categorical variables and Mann–Whitney U tests for continuous variables

**P < 0.001; *P < 0.05

### Abnormal Domains of Children with SDD

Of the 2733 SDD children with attributes in several of the abnormal developmental domains, most of them (62.2%) had only one abnormal domain, the most common one being gross motor skills problems (51%). In addition, it was found that over one-tenth of children SDD (11.1%) had problems in all of the domains (Table 2).

**Table 2:** Children with delayed development by abnormal domain (N = 2733)

Number of abnormal domains	Abnormal domain	n	(%)
1	GM	1393	(51)
	FM	107	(3.9)
	RL	38	(1.4)
	EL	114	(4.2)
	PS	48	(1.8)
	Total	1700	(62.2)
2	GM + FM	259	(9.5)
	GM + RL	22	(0.8)
	GM + EL	75	(2.7)
	GM + PS	47	(1.7)
	FM + RL	9	(0.3)
	FM + EL	16	(0.6)
	FM + PS	17	(0.6)
	RL + EL	33	(1.2)
	RL + PS	6	(0.2)
	EL + PS	14	(0.5)
	Total	498	(18.2)
3	GM + FM + RL	44	(1.6)
	GM + FM + EL	29	(1.1)
	GM + FM + PS	32	(1.2)
	GM + RL + EL	7	(0.3)
	GM + RL + PS	5	(0.2)
	GM + EL + PS	17	(0.6)
	FM + RL + EL	13	(0.5)
	FM + RL + PS	6	(0.2)
	FM + EL + PS	6	(0.2)
	RL + EL + PS	7	(0.3)
	Total	166	(6.1)
4	GM + FM + RL + EL	18	(0.7)
	GM + FM + RL + PS	7	(0.3)
	GM + FM + EL + PS	18	(0.7)
	GM + RL + EL + PS	5	(0.2)
	FM + RL + EL + PS	17	(0.6)
	Total	65	(2.4)
5	GM + FM + RL + EL + PS	304	(11.1)
	Total	304	(11.1)

*GM* gross motor skills, *FM* fine motor skills, *RL* receptive language skills, *EL* expressive language skills, *PS* personal, and social skills

### Risk Factors for SDD

Table 3 reports the univariable logistic regression analysis results for the potential risk factors of DD in the high-risk children under 5 years of age. Children with a gestational age at birth of less than 37 weeks (odds ratio [OR] = 1.62; 95% confidence interval [CI] = 1.48–1.78; *P* < 0.001) and/or a birth weight of less than 2500 g (OR 1.58; 95% CI 1.35–1.86; *P* < 0.001) along with a mother who was not a government officer (OR 1.52; 95% CI] = 1.18–1.96; *P* = 0.0007), had a low educational level (OR 1.50; 95% CI 1.33–1.69; *P* < 0.001), was a non-smoker (OR 1.71; 95% CI 1.00–2.93; P = 0.039), had poor nutritional status (OR 1.47; 95% CI 1.22–1.78; *P* < 0.001), and/or lived at a high altitude (OR 1.72; 95% CI 1.45–2.05; *P* < 0.001) had a significantly higher risk of SDD.

**Table 3:** Univariable logistic regression analysis of risk factors for suspected developmental delay

Variable	n/N (%)	OR (95%CI)	*P* value
Mother's occupation			< 0.001**
Government officer	71/538 (13%)	1.00	
Others	2394/12,741 (19%)	1.52 (1.18–1.96)	
Mother's education			< 0.001**
Primary school	426/1738 (25%)	1.50 (1.33–1.69)	
Higher than primary school	2058/11,552 (18%)	1.00	
Marital status			0.285
Civil union	2268/12,280 (18%)	1.00	
Others	107/526 (20%)	1.13 (0.91–1.40)	
Number of pregnancies			0.922
1	1256/6763 (19%)	1.00 (0.92–1.10)	
≥ 2	1239/6695 (19%)	1.00	
Smoking status			0.039*
No	2718/14,191 (19%)	1.71 (1.00–2.93)	
Yes	15/123 (12%)	1.00	
Poor nutritional status			< 0.001**
No	2582/13,720 (19%)	1.00	
Yes	151/594 (25%)	1.47 (1.22–1.78)	
Heavy alcohol consumption			0.668
No	2728/14,283 (19%)	1.23 (0.47–3.20)	
Yes	5/31 (16%)	1.00	
Illicit drug use			0.582
No	2726/14,270 (19%)	1.25 (0.56–2.80)	
Yes	7/44 (16%)	1.00	
High altitude			< 0.001**
No	2542/13,639 (19%)	1.00	
Yes	191/675 (28%)	1.72 (1.45–2.05)	
Had an adverse pregnancy outcome			0.394
No	1721/8912 (19%)	1.04 (0.95–1.13)	
Yes	1012/5402 (19%)	1.00	
Gestational age at birth			< 0.001**
< 37 weeks	1350/6162 (22%)	1.62 (1.48–1.78)	
≥ 37 weeks	988/6698 (15%)	1.00	
Birth weight			< 0.001**
< 2500 g	2298/11,868 (19%)	1.58 (1.35–1.86)	
≥ 2500 g	187/1421 (13%)	1.00	

*n* number of children with suspected developmental delay,

*N* number of children; OR, odds ratio

**P < 0.001; *P < 0.05

According to the results of the multivariable analysis, all of the significantly associated factors in the univariable analysis were also associated with SDD in the high-risk children, except for the smoking behavior of the mother (*P* = 0.106). High-risk children with a gestational age at birth of < 37 weeks (adjusted OR [aOR] 1.54; 95% CI 1.39–1.70; *P* < 0.001) and/or a birth weight of < 2500 g (aOR 1.22; 95% CI 1.02–1.45; *P* < 0.001) along with a mother who was not a government officer (aOR 1.46; 95% CI 1.11–1.93; *P* = 0.007), with low education level (aOR 1.36; 95% CI 1.19–1.55; *P* < 0.001), with poor nutritional status (aOR 1.34; 95% CI 1.09–1.65; *P* = 0.005), and/or living in a high-altitude area (aOR 1.59; 95% CI 1.32–1.91; *P* < 0.001) were at a higher risk of SDD (Table 4).

**Table 4:** Multivariable logistic regression analysis of the risk factors for suspected developmental delay

Variable	aOR (95% CI)	*P* value
Non-government officer mother	1.46 (1.11–1.93)	0.007*
Low education level mother	1.36 (1.19–1.55)	< 0.001**
Non-smoking mother	1.57 (0.91–2.71)	0.106
Poor nutritional status mother	1.34 (1.09–1.65)	0.005*
Mother lives at a high altitude	1.59 (1.32–1.91)	< 0.001**
Gestational age at birth < 37 weeks	1.54 (1.39–1.70)	< 0.001**
Birth weight < 2500 g	1.22 (1.02–1.45)	< 0.001**

*aOR* adjusted odds ratio, *CI* confidence interval

**P < 0.001; *P < 0.05

## Discussion

In this cross-sectional study of high-risk children in Thailand under 5 years of age with a birth weight of less than 2500 g and/or birth asphyxia, we found that the prevalence of SDD was 19.1%, which is much higher than the 6.7% previously evaluated using the combined indicator early childhood development index (ECDI) on children aged 36–59 months old in Thailand (Gil et al., 2020). Since the domains considered in the ECDI (physical, social-emotional, learning, and literacy-numeracy) are different to those in the DAIM, the difference in SDD prevalence could be due to considering different criteria of the indicators using each tool. Even though there was a substantial number of children with a low birth weight and/or asphyxia during birth who did not show DD, they still need to be monitored because the condition might show up later. The results of previous studies in Thailand indicate that the prevalence of DD in Thai children aged eight months to 12 years old assessed using the Denver II tool in 2014 (27.2%) (Losatiankit et al., 2017) and aged twelve months old assessed using the DAIM in 2022 (30.2%) (Ratanatharathorn et al., 2022) were higher than the SDD prevalence in our study. An SDD prevalence lower than the DD prevalence infers that some incidences of DD could show up later on after the first assessment. In addition to monitoring, strategies to outreach people in underserved regions such as high-altitude areas (e.g., primary screening by community health workers or the parents) should be considered. There is a need for both prenatal and postpartum support to ensure access to preventative care and treatment to prevent preterm births and birth complications, and to support early infancy and developmental screening.

Low maternal educational level (primary school or lower), poor maternal nutritional status, short gestation period before birth (< 37 weeks), and low infant birth weight (< 2500 g) were high-risk factors for SDD, which is in agreement with the findings of other studies (Halpern et al., 2000; Sitaresmi et al., 2016; Zhang et al., 2018). A child whose mother was not a government officer was also found to be at a higher risk of SDD in our study. This is consistent with the findings of a previous study in Thailand (Losatiankit et al., 2017), in which children whose mothers were farmers had a twofold higher risk of DD than those whose mothers were government officers. The author also suggested that a well-paid and stable occupation such as being a government officer was linked to the well-being of the mother and the child. In Thailand, an occupation in government service is stable with a fixed period of work (Permsuwan, 2010), so mothers who are government officers can spend more time after work caring for their children. Moreover, they are less concerned about the future as they have a steady income and are seldom laid off, as happens with most other occupations in Thailand.

We found a higher risk of SDD among high-risk children with mothers living at a high altitude. The highlands, which accounts for 53% of the land area in 20 provinces (Chiang Mai, Chiang Rai, Mae Hong Son, Phayao, Lamphun, Phrae, Nan, Lampang, Tak, Phetchabun, Phitsanulok, Loei, Sukhothai, Kamphaeng Phet, Kanchanaburi, Uthai Thani, Suphanburi, Ratchaburi, Prachuap Khiri Khan, and Phetchaburi) contain the residential areas of various hill tribes. Most of the highland villages (88%) are relatively inaccessible, thereby making it difficult for government agencies to operate efficiently. For this reason, the people living there suffer from a lack of education, healthcare, and nutrition (National Research Council of Thailand, 2016; Suwannasuan et al., 2013). Moreover, the highlands in the northern region experience severe particulate matter (PM_2.5_) problems each year (Pollution Control Department, 2017). Exposure to air contamination has been connected to decelerated neurological development early in life and an increased risk of neurodevelopmental problems in children. In addition, a previous study in schoolchildren found that prenatal air pollution exposure might harm the brain structure (Sunyer & Dadvand, 2019). It would be interesting to determine whether PM_2.5_ directly causes DD in children.

The results of our univariable analysis show an increased risk of SDD in children with non-smoking mothers, which is inconsistent with those in previous studies in which maternal smoking was found to be linked with the risk of abnormal development in children (Julvez et al., 2007; Slykerman et al., 2007). In addition, this variable was eliminated from the multivariable model after adjusting for other potential risk factors. The sample size of mothers who smoked was very small (*n* = 123) compared to non-smoking mothers (*n* = 14,191). This difference could have resulted in a different direction of association in the univariable model and subsequent elimination from the multivariable model.

Although researchers have previously reported that maternal alcohol assumption has a negative effect on child development, (Hagan et al., 2016; Yoshida et al., 2018) we found that it was not associated with a high risk of DD in our study. Like the issue of sample size for the smoking status variable, this inconsistency could have resulted from the small sample size of mothers who drinking alcohol (*n* = 36) compared to non-drinkers (*n* = 14,283). However, it might be interesting to investigate the frequency of alcohol drinking during pregnancy in a future study.

A major limitation of the present study could be not including several factors that have previously been associated with a high risk of SDD, such as gestational diabetes, the inter-pregnancy interval, breastfeeding, previous hospital admission of the children, parental activities for enhancing speech and language skills, and a lack of literature suitable for children at home (de Moura et al., 2010; Halpern et al., 2000; Sharma et al., 2019). Some of the clinical assessments during pregnancy such as gestational diabetes were not available at the time of data collection, and so a future study in which these known potential risk factors are accounted for might be interesting.

The strength of this study is that this is the first time of using data on children from across Thailand, while several previous studies on child development in Thailand included data only from some settings (Jundaboot et al., 2018; Kanpirom & Masena, 2017; Saengjun, 2017; Vuttipittayamongkol, 2018). Indeed, the large sample size covering several settings across the country would be useful for further study on or developing strategies to SDD monitoring or prevention of DD in Thailand.

## Conclusions

The findings of this study reveal that maternal (occupations and educational level), infant (birth weight, gestational age at birth, and nutrition status), and environment (living in a high-altitude area) factors were associated with a high risk of SDD. These findings highlight the usefulness of the early identification of children at a high risk of SDD, which can lead to them accessing appropriate stimulation at an earlier stage in their development. SDD monitoring of children by community health workers and/or by developing outreach strategies, especially in underserved regions, should be considered. In addition, developing policies and guidelines, and intervention for high-risk children ought to be conducted to reduce the subsequent problems caused by the late detection of DD.

## Data Availability

Data are available upon reasonable request.
